# Association between objectively evaluated physical activity and sedentary behavior and screen time in primary school children

**DOI:** 10.1186/s13104-017-2495-y

**Published:** 2017-05-02

**Authors:** Chiaki Tanaka, Maki Tanaka, Masayuki Okuda, Shigeru Inoue, Tomoko Aoyama, Shigeho Tanaka

**Affiliations:** 1 Division of Integrated Sciences, J. F. Oberlin University, 3758 Tokiwamachi, Machida, Tokyo, 194-0294 Japan; 2 Department of Child Education, Kyoto Seibo College, 1 Taya-cho, Fukakusa Fushimi-ku, Kyoto, 612-0878 Japan; 3 Department of Environmental Medicine, Graduate School of Science and Engineering, Yamaguchi University, 1-1-1 Minamikogushi, Ube, 755-8505 Japan; 4 Department of Preventive Medicine and Public Health, Tokyo Medical University, 6-1-1 Shinjuku, Shinjuku-ku, Tokyo, 160-8402 Japan; 5 Department of Nutrition and Metabolism, National Institute of Health and Nutrition, National Institutes of Biomedical Innovation, Health and Nutrition, 1-23-1 Toyama, Shinjuku-ku, Tokyo, 162-8636 Japan

**Keywords:** Exercise, Ambulatory activity, Non-ambulatory activity, Sitting, Accelerometer, Screen time

## Abstract

**Background:**

Even when meeting guidelines for physical activity (PA), considerable sedentary time may be included. This study in primary school children investigated the relationships between objectively evaluated sedentary and PA times at different intensities using triaxial accelerometry that discriminated between ambulatory and non-ambulatory PA. The relationships between subjectively evaluated screen time (i.e. time spent viewing television and videos, playing electronic games, and using personal computers) and objectively evaluated sedentary and PA times were examined.

**Methods:**

Objectively evaluated sedentary and PA times were assessed for 7 consecutive days using a triaxial accelerometer (Active style Pro: HJA-350IT) in 426 first to sixth grade girls and boys. Metabolic equivalents [METs] were used to categorize the minutes of sedentary time (≤1.5 METs), light PA (LPA, 1.6–2.9 METs), moderate-to-vigorous PA (MVPA,  ≥3.0 METs) and vigorous PA (VPA,  ≥6.0 METs). The physical activity level (PAL) was calculated using the mean MET value. Subjectively evaluated screen time behaviors were self-reported by participants and parents acting together. The associations between PA and sedentary and screen time variables were examined using partial correlation analyses.

**Results:**

After adjustment for age, body weight and wearing time, objectively evaluated sedentary time correlated strongly with non-ambulatory and total LPA and PAL, moderately with ambulatory LPA, non-ambulatory or total MVPA, and weakly with ambulatory MVPA, ambulatory, non-ambulatory or total VPA. Subjectively evaluated screen time was not associated significantly with objectively evaluated sedentary and PA times or PAL. On average, each reduction of 30 min in daily sedentary time was associated with 6 or 23 min more of MVPA or LPA, respectively.

**Conclusions:**

These findings show that higher daily sedentary time may be compensated mainly by lower LPA, while the association between sedentary time and MVPA was moderate. Therefore, improving MVPA and reducing sedentary time are important in primary school children.

## Background

The World Health Organization guidelines on physical activity (PA) recommend at least 60 min every day of moderate-to-vigorous physical activity (MVPA) in order to benefit the health of children and adolescents [1]. However, considerable sedentary time can be accumulated even when these PA guidelines are met. For example, Marshall et al. [2] reported that clusters of UK and USA adolescents of both genders had higher than average levels of PA, but also increased levels of screen-based sedentary behavior or sedentary socializing activities. Sedentary behavior is usually defined as behaviors carried out in a seated or reclining position with an energy cost of ≤1.5 METs [3]. A previous systematic review of the relationship between sedentary time or sedentary behavior and health-related outcomes in children and adolescences showed that long periods of sedentary activity were associated with adverse health outcomes [4–7]. Recently, guidelines for sedentary behavior in children and adolescents have been published in several countries [8–10].

Whether or not more time spent in MVPA is associated with higher physical activity level (PAL) remains an interesting question. One of the first studies to examine this possibility was carried out in adults [11]. A multiple regression analysis of the proportion of time spent on moderate or high intensity activities showed that only moderate intensity activity was a significant predictor of PAL (r^2^ = 0.51), while the proportions of low and moderate intensity activities influenced total energy expenditure. Recently, Pearson et al. [12] reviewed several observational studies that examined the association between PA and sedentary behavior or sedentary time in young people (<18 years), and showed only a weak negative association. However, only a small number of studies have examined the associations between objectively evaluated PA and sedentary time [13].

Previous studies proposed using prediction models of metabolic equivalents (METs) for children with accelerometers. The slope and intercept of ambulatory activities in a predictive model such as walking and running are different from those of non-ambulatory activities, like playing games, cleaning, playing with blocks, tossing a ball, and aerobic dance [14–17]. Based on the variability in accelerometer counts, Hikihara et al. [18] showed a discrimination between ambulatory activities, such as continuous walking or jogging and non-ambulatory activities, including lifestyle activity. Our previous study in primary school children reported that ambulatory light PA (LPA) and MVPA and non-ambulatory LPA were lower in the summer vacation than during the school year in both genders [19]. We also observed that non-ambulatory MVPA in girls was significantly lower during the summer vacation than in the school year. Another study in preschool children showed that 25 m run speed, skill-related physical fitness total Z-score, and total physical fitness Z-score (health-related and skill-related physical fitness total Z-score) correlated positively with the time spent in ambulatory activity [20]. Moreover, thin preschool children spent significantly less time engaged in ambulatory PA than normal-weight or overweight children [21]. Although the contribution of ambulatory and non-ambulatory PAs to health outcomes in children has not been well established [20, 21], physical fitness and weight status in children correlated to the types of PA, MVPA was shown to make up approximately 40–45% of non-ambulatory activity in primary school boys and girls in the school year [19]. Therefore, ambulatory and non-ambulatory PAs may have different effects on the relationships between PAs and sedentary behavior (SB). The objective of the present study was to examine the association between PA intensities and sedentary time in 6–12 year-old Japanese primary school children, using objective accelerometer data for PA that discriminated between non-ambulatory or ambulatory PA. We also examined the contribution of reported screen time to total sedentary and PA times.

## Methods

Our convenience sample included Japanese primary school children from 14 primary schools in urban areas of Tokyo and Kyoto. The participants were invited to participate in the study at their school using leaflets, such as a newsletter. Informed consent was obtained from all participants, and the Ethical Committee of J. F. Oberlin University approved the study protocol (Receipt Number: 12023). All participations and their parents consented to publication of the data. The data of anthropometric measurements, sedentary time, and PA were collected from June 2012 to January 2015 during the school year. The initial sample comprised 569 participants with 143 children subsequently being withdrawn because of: accelerometer data not conforming with the study criteria (see below) [n = 95], revocation of the agreement [n = 7], history of conditions affecting PA such as respiratory disease or heart disease [n = 25], no questionnaire data [n = 15], and belonging to a different ethnic group [n = 1]. Data analysis was carried out on the remaining 426 children. These children, except those who revoked the agreement [n = 7], participated in the study, but their data were excluded from the analyses. There was no significant difference in age, relative weight and gender proportion between the study group and children withdrawn from the analyses.

### Objective measurement of sedentary time and physical activity

Habitual sedentary time and PA were measured using a triaxial accelerometer (Active style Pro HJA-350IT, Omron Healthcare, Kyoto; dimensions 74 × 46 × 34 mm and weight 60 g including batteries). The device is described in detail elsewhere [18]. The participants wore the accelerometer on the left side of the waist at school. We calculated the synthetic acceleration in all three axes using signals before and after high-pass filtering. The ratio of unfiltered to filtered acceleration was then calculated to identify non-ambulatory activities (e.g., playing games, playing with blocks, tossing a ball, and cleaning and clearing away) and ambulatory activities (e.g., walking and running). When the ratio of unfiltered to filtered synthetic acceleration was 1.12, the rate of correct discrimination between non-ambulatory and ambulatory activities was excellent, with a mean of 99.1% [18]. The acceleration signals were calculated as the mean of the absolute values of accelerometer output in each axis over 10 s epochs at the middle of each activity. The data were expressed as variables of acceleration output. Because the predictive equations used for the Active style Pro were established in adults, the values of metabolic equivalent (MET) values recorded by the accelerometer are overestimated in primary school children [18]. We therefore used the following conversion equations for primary school children, based on the results of Hikihara et al. [18].

$${\text{Ambulatory activities:}} \, 0. 6 2 3 7 \times {\text{MET value of Active style Pro}} + 0. 2 4 1 1$$

$${\text{Non-ambulatory activities:}} \, 0. 6 1 4 5 \times {\text{MET value of Active style Pro}} + 0. 5 5 7 3$$

Sedentary time and PA were monitored continuously for 7 days. The participants were requested to wear the devices at all times, except under special circumstances, such as dressing and bathing. In fact, many participants wore the accelerometer during sleep. Because sleep and sedentary time cannot be discriminated we analyzed data collected between 7:00 and 21:00 to exclude sleep time. We included days in which >600 min (10 h) of wearing time had accrued. Periods with >60 min of consecutive ‘‘non-wearing time’’ were as classified as non-wearing time. Penpraze et al. [22] and Cliff et al. [23] suggested at least 3 days were required for reliable PA monitoring in young children. Participants with data from at least 2 weekdays and at least 1 weekend day were included in the analysis.

### Self-reported measures

Time spent viewing television and videos, playing electronic games and using a personal computer was assessed by a questionnaire completed jointly by the children and their parents. The children and parents were asked the following two questions: “How many hours of television and video movies (except at school) does the child usually watch? (a) in a school day or (b) in a non-school day—0, 30 min, 1, 2, 3, 4, or >5 h; and “How many hours in a single day does the child usually use a personal computer or play electronic games (including television, personal computer, portable game machines such as mobile phones at home and friends’ homes including arcade games, etc.)? (a) in a school day or (b) a non-school day—0, 30 min, 1, 1.5, 2, 2.5, or >3 h.

### Anthropometric measurements

Body height and weight were measured without shoes, but with clothing to the nearest 0.1 cm and 0.1 kg, respectively. Net body weight was calculated as the weight of clothing subtracted from the measured body weight. BMI (body mass index) was calculated as weight in kilograms divided by height in meters squared. Weight status was classified as normal weight, overweight/obese, or thin using the Japanese cut-offs for weight based on national reference data for Japanese children [24].

Relative weight was calculated as [body weight (kg) − standard weight for gender, age, and height (kg)]/standard weight (kg) × 100 (%)

$$*\,{\text{standard weight (kg)}} = {\text{a}} \times {\text{measured body height (cm)}} - {\text{b}}$$

a and b are gender- and age-specific.

The cut-off values for weight categories were: overweight/obesity combined, ≥ +20%; normal weight, −20 to +20%; thin, ≤ −20%.

### Analyses

The time spent in sedentary and each PA intensity each day was calculated using METs for each individual: average number of weekday and weekend minutes spent in sedentary time (METs ≤1.5), LPA (METs 1.6–2.9), MVPA (METs ≥3.0) and VPA (METs ≥6.0). The mean weekly values were then calculated. Time spent viewing TV and video games was calculated as (1) 0 min, (2) 30 min, (3) 1 h, (4) 2 h, (5) 3 h, (6) 4 h, (7) 5 h. Time using a personal computer, or playing electronic games was calculated as (1) 0 min, (2) 30 min, (3) 1 h, (4) 1.5 h, (5) 2 h, (6) 2.5 h, or (7) 3 h. For the objective and subjective data, the mean values were calculated by weighting for 5 weekdays and 2 weekend days [Weighted data = ((mean for weekdays × 5) + (mean for weekend days × 2))/7]. PA assessed by the accelerometer was presented as: (1) PA states for ambulatory activity or non-ambulatory activity in intensity-specific categories (LPA, MVPA, and VPA); and (2) PAL, total energy expenditure (kcal/day) divided by basal metabolic rate (kcal/day), calculated using the mean value of METs.

The relationship between two variables was examined using partial correlation analysis controlled for gender, age, body weight, and wearing time. A simple linear stepwise regression analysis was used to obtain prediction equations. The dependent variables were sedentary time, LPA, and MVPA and the independent variables sedentary time, LPA, MVPA or VPA, and gender, age, body weight, and wearing time. The results are shown as mean ± standard deviation (SD). The statistical analyses were performed using IBM SPSS statistics 20.0 for Windows (IBM Co., Tokyo, Japan). All statistical tests were regarded as significant when *p* values were ≤0.05.

## Results

### Characteristics of the study participants

Table 1 shows the characteristics of the study participants and time spent at sedentary, different intensity levels and total time for ambulatory and non-ambulatory activity, PAL, and subjectively evaluated screen-based sedentary behavior. Five percent of participants were overweight/obese. The duration of accelerometry was considerably greater than the minimum criteria specified (at least 3 days and 10 h), with a mean of 6.3 days and 13.4 h, respectively. The percentage of children with >2 h/day of screen time was 59.8%. The percentages of each response category (1–7 corresponding to 0, 30 min, 1, 2, 3, 4, or >5 h, respectively) for television and video viewing time in a school or non-school day were 6.9% (school day) and 0.7% (non-school day) in category 1; 13.8 and 2.4% in category 2; 33.1 and 14.3% in category 3; 33.1 and 32.6% in category 4; 9.3 and 26.9% in category 5; 2.1 and 14.5% in category 6; and 1.7 and 8.6% in category 7. The corresponding percentages in games and personal computer time (1–7 corresponding to 0, 30 min, 1, 1.5, 2, 2.5, or >3 h, respectively) were 46.1% (school day) and 25.1% (non-school day) in category 1; 28.2 and 25.8% in category 2; 15.5 and 22.9% in category 3; 6.0 and 6.9% in category 4; 3.6 and 11.9% in category 5; 0.2 and 2.1% in category 6; and 0.5 or 5.3% in category 7.

**Table 1:** Physical characteristics and determinants at baseline of the participants

Variables	n = 426	
	Average	SD
Age (year)	9.3 ± 1.6	
Height (cm)	132.3 ± 10.7	
Weight (kg)	29.0 ± 7.5	
Body mass index (kg/m^2^)	16.3 ± 2.2	
Relative weight (%)	−2.9 ± 11.9	
Sedentary behaviour (min/day)	363 ± 62	
LPA (min/day)		
Ambulatory	105 ± 19	
Non-ambulatory	225 ± 43	
Total time	360 ± 50	
MVPA (min/day)		
Ambulatory	39 ± 15	
Non-ambulatory	28 ± 9	
Total time	68 ± 21	
VPA (min/day)		
Ambulatory	5 ± 3	
Non-ambulatory	1 ± 1	
Total time	6 ± 4	
Physical activity level	1.74 ± 0.07	
Wearing time (min/day)	802 ± 34	
Wearing day (days)	6.3 ± 1.0	
Television and video viewing time (min/day) (n = 420)	110 ± 60	
Time spent playing electronic games and using personal computers (min/day) (n = 419)	36 ± 37	
Total screen time (min/day) (n = 415)	146 ± 80	

*LPA* light physical activity, *MVPA* moderate-to-vigorous physical activity, *VPA* vigorous physical activity

Table 2 shows the partial correlation between duration of objectively evaluated sedentary time, each intensity and type of PA, PAL, and screen time. After adjustment for age, gender, body weight, and wearing time, objectively evaluated sedentary time correlated strongly with non-ambulatory and total LPA and PAL, moderately with ambulatory LPA, non-ambulatory and total MVPA, and weakly with ambulatory MVPA and ambulatory, non-ambulatory and total VPA. Screen time was not associated significantly with objectively evaluated sedentary time, PAs, or PAL.

**Table 2:** Partial correlation coefficients between daily sedentary behavior and physical activity

Variables	Sedentary time (min/day)	LPA (min/day)	MVPA (min/day)	VPA (min/day)	Physical activity level
Sedentary time (min/day)	–	–	–	–	−0.87*
LPA (min/day)					
Ambulatory	−0.58*	0.49*	0.44*	0.24*	0.55*
Non-ambulatory	−0.81*	0.91*	0.18*	−0.04	0.52*
Total time	−0.95*	–	0.34*	0.06	0.68*
MVPA (min/day)					
Ambulatory	−0.41*	0.13*	0.90*	0.68*	0.74*
Non-ambulatory	−0.66*	0.53*	0.72*	0.26*	0.73*
Total time	−0.61*	0.34*	–	0.62*	0.89*
VPA (min/day)					
Ambulatory	−0.24*	0.05	0.58*	0.97*	0.58*
Non-ambulatory	−0.26*	0.08	0.57*	0.82*	0.57*
Total time	−0.27*	0.06	0.62*	–	0.62*
Television and video viewing time (min/day) (n = 420)	0.02	−0.06	0.09	0.04	0.02
Time spent playing electronic games and using personal computers (min/day) (n = 419)	0.06	−0.06	−0.02	−0.03	−0.05
Total screen time (min/day) (n = 415)	0.05	−0.08	0.04	0.01	−0.02

Adjusted variables: age, sex, body weight and wearing time

*LPA* light physical activity, *MVPA* moderate-to-vigorous physical activity, *VPA* vigorous physical activity

* p < 0.05

The results of the stepwise linear regression analysis (Table 3) showed that on average, each additional 10 min each day of MVPA or LPA was associated with 18 and 12 min less of objectively evaluated sedentary time, respectively. In contrast, each 30 min reduction in daily sedentary time was associated with 6 or 23 min more of objectively evaluated MVPA or LPA.

**Table 3:** Prediction equations for sedentary behavior or moderate-to-vigorous physical activity

Prediction equations	Adjusted R^2^	SEE	p value
Sedentary behavior (min/day) = −64 (50) − 1.83 (0.12) * MVPA (min/day) + 0.606 (0.057) * wearing time (min/day) − 19.7 (4.4) * sex + 6.88 (1.74) * age (year) + 1.06 (0.35) * body weight (kg)	0.60	39.6	<0.001
Sedentary behavior (min/day) = −111 (19) − 1.16 (0.02) * LPA (min/day) + 1.04 (0.02) * wearing time (min/day) + 18.5 (1.6) * sex + 3.30 (0.55) * age (year)	0.94	15.7	<0.001
Sedentary behavior (min/day) = −271 (59) − 3.75 (0.59) * VPA (min/day) + 0.60 (0.07) * wearing time (min/day) + 15.48 (2.05) * age (year) + 1.13 (0.44) * body weight (kg)	0.40	48.5	<0.001
LPA (min/day) = −71 (16) − 0.78 (0.01) * sedentary behavior (min/day) + 0.85 (0.02) * wearing times (min/day) + 15.3 (1.3) * sex + 1.22 (0.47) * age (year)	0.93	12.9	<0.001
LPA (min/day) = 56 (49) + 0.84 (0.11) * MVPA (min/day) + 0.38 (0.06) * wearing time (min/day) + 20.3 (4.3) * sex − 6.61 (1.71) * age (year) − 1.04 (0.35) * body weight (kg)	0.39	38.8	<0.001
MVPA (min/day) = 67 (16) − 0.20 (0.01) * sedentary behavior (min/day) + 0.14 (0.02) * wearing time (min/day) − 14.9 (1.3) * sex − 1.16 (0.48) * age (year)	0.59	13.2	<0.001
MVPA (min/day) = 78 (10) + 0.13 (0.02) * LPA (min/day) − 18.2 (1.6) * sex − 3.28 (0.55) * age (year)	0.41	15.8	<0.001

*SEE* standard error of estimate, sex: boy: 1, girl: 2, LPA light physical activity, *MVPA* moderate-to-vigorous physical activity, *VPA* vigorous physical activity, the values given in parentheses in prediction equations were SEE for each partial regression coefficient

## Discussion

This study examined the associations between sedentary time and PAL or PAs classified as either ambulatory or non-ambulatory using triaxial accelerometry in primary school Japanese children. We showed significant associations between objectively evaluated sedentary time and PAL or PAs. In particular, non-ambulatory and total LPA correlated strongly with sedentary time, with the increase in sedentary time being compensated mainly by decreased LPA. On the other hand, there was only a moderate degree of correlation between MVPA and sedentary time, while no association was found between subjectively evaluated screen time and objectively evaluated sedentary time, PAs or PAL. These findings indicate that improvement in MVPA and decrease in sedentary time may be independent of each other to some degree in primary school children.

PAL for level II (moderate) and level III (high) in Japanese individuals is categorized as 1.60 and 1.80 for 8–9 years and 1.65 and 1.85 for 10–11 years, respectively. These Dietary Reference Intakes for Japanese—2015—are based on the doubly labeled water method [25]. The mean value of PAL (1.74) in the sample of the present study (9.3 ± 1.6 years) was categorized as level II (moderate). Screen time over 2 h/day was found in 59.8% of children. Recently, Pearson et al. [12] reviewed several observational studies that had examined the association between PA and objectively evaluated sedentary behavior or screen time in young people (<18 years), and showed only a small, negative association. This suggested that these behaviors did not directly displace one another.

Studies to date have assessed the association between objectively measured PAL, PA intensities, and sedentary time. Several studies [26–29] showed relatively strong and negative correlations between sedentary time or low-intensity PA and total PA such as PAL. On the other hand, some studies reported a negative correlation between sedentary time and MVPA [13, 28, 29], whereas others reported no such association [26, 27]. These discrepancies were probably due to differences in sample characteristics, accelerometry, and cutoffs between sedentary time, LPA, and MVPA. Our results are in agreement with some of these previous studies in children and adolescents. When interpreting the differences in results between studies in children and adolescents, differences in the definition or algorism of MVPA and differences in epoch length between studies may have contributed to the conflicting results [30–33]. In contrast to the findings in adults, where PAL can be increased by increasing the amount of time spent on moderate intensity activities and reducing low-intensity activities [11], children and adolescents are characterized by short, intermittent bouts of VPA [34, 35]. The association between sedentary time and MVPA observed in the present study was not seen in previous studies. The associations between sedentary time and MVPA were not significantly different between boys and girls and lower grade and upper grade individuals (1–3 grade boys, −0.64; 1–3 grade girls, −0.58; 4–6 grade boys, −0.59; 4–6 grade girls, −0.68). Therefore, all children were included in our analyses.

In the present study, time spent on screen-based sedentary behavior (TV and video viewing time, PC and game time, and total screen time) recorded in the questionnaire did not correlate significantly with sedentary time assessed by the accelerometer. Lubans et al. [36] reviewed the validity of self- and proxy-report measures of sedentary behavior estimates of screen time to assess the utility of accelerometers to classify sedentary time in children and adolescents and showed self-reported measures remain largely untested [36]. In the present study, PAs and PAL were not associated with screen time. Herman et al. [13] also showed that neither MVPA nor LPA were associated with screen time in children. Self- and proxy-report measures of sedentary behavior instead provided information about the type of sedentary behavior or context. Studies on the association between screen time and PAs are therefore needed to further understand the complex relationships between sedentary behavior and PAs.

The present study indicated that considerably more time was spent in non-ambulatory LPA than ambulatory LPA. On the other hand, non-ambulatory and ambulatory MVPA minutes were comparable. Previous studies did not, however, discriminate between ambulatory and non-ambulatory PA. Objectively evaluated sedentary time showed a stronger negative correlation with non-ambulatory MVPA or LPA (r = −0.66 and −0.81, respectively) than ambulatory MVPA or LPA (r = −0.41 and −0.58, respectively). Although there is only limited evidence on the association between ambulatory and non-ambulatory PAs and health outcome in children [20, 21], physical fitness and weight status in children seem to be related to the types of PA. MVPA represents approximately 40–45% of non-ambulatory activity in primary school boys and girls during the school year [19]. Evidence from the current study also indicates that both ambulatory activity and non-ambulatory activity are important factors in the PA lifestyle of children. Therefore, public health strategies should target LPA and non-ambulatory MVPA to decrease sedentary time and improve overall PA and health in children. These data may be particularly important for providing insights into improving different intensities of PA in children. On average, each additional 10 min of daily MVPA or LPA was associated with 18 and 12 min less of objectively evaluated sedentary time. Over the course of a week, sedentary time was compensated mainly by higher daily LPA, as indicated by the strong correlation and partial regression coefficient being close to 1.0. Moreover, although the correlation between MVPA and sedentary time was slightly weaker, higher daily MVPA may lead to lower sedentary time and higher total PA to some degree, probably due to concomitant higher levels of LPA. In contrast, each 30 min reduction in daily sedentary time was associated with 6 or 23 min more of objectively evaluated MVPA or LPA. Therefore, the discrepancy in the corresponding durations was larger in the latter case (18 and 12 min vs. 6 and 23 min for MVPA and LPA, respectively). The main reason for this observation may be that reduced sedentary time was displaced mainly by LPA and not MVPA, while higher MVPA accompanied LPA, leading to sufficiently lower sedentary time.

There are several methodological points that need to be considered when interpreting our results. Firstly, our sample was not a representative sample of Japanese children. Secondly, the accelerometer is a widely used tool to measure PA, but it cannot assess all PA, such as swimming and cycling. These two points were limitations of the study. The strengths of our study include the use of objective and quantitative measures of sedentary time, classifying ambulatory and non-ambulatory PA, and the use of a sample population of Japanese primary school children from 14 different schools. It should be noted that the data were recorded over a 10-sec epoch, which should be sufficiently sensitive to pick up short bursts of vigorous activity [31, 37]. Shorter epoch lengths could be used to better reflect movement patterns of children.

## Conclusions

In Japanese primary school children, objectively evaluated sedentary time correlated strongly with non-ambulatory and total LPA or PAL, moderately with ambulatory LPA, non-ambulatory or total MVPA, and weakly with ambulatory MVPA, ambulatory, non-ambulatory or total VPA, after adjustment for age, gender, body weight and wearing time. Screen time was not associated significantly with objectively evaluated sedentary time, PAs, or PAL. On average, each additional 10 min of daily MVPA or LPA was associated with 18 or 12 min less of objectively evaluated sedentary time. In contrast, each 30 min reduction in daily sedentary time was associated with 6 or 23 min more of objectively evaluated MVPA or LPA. These findings indicate that higher daily sedentary time was compensated by lower LPA, with only a moderate association between sedentary time and MVPA. Taken together, these findings indicate that evaluation of non-ambulatory activity or LPA is important in the overall assessment of PA.

## Abbreviations

PA: physical activity
SB: sedentary behavior
METs: metabolic equivalents
LPA: light intensity activity
MVPA: moderate-to-vigorous physical activity
VPA: vigorous physical activity
PAL: physical activity level
BMI: body mass index
TV: television
